# Energy-Efficient Depth-Based Opportunistic Routing with Q-Learning for Underwater Wireless Sensor Networks

**DOI:** 10.3390/s20041025

**Published:** 2020-02-14

**Authors:** Yongjie Lu, Rongxi He, Xiaojing Chen, Bin Lin, Cunqian Yu

**Affiliations:** 1 College of Information Science and Technology, Dalian Maritime University, Dalian 116026, China; yongjielu2017@dlmu.edu.cn (Y.L.); yucunqian@dlmu.edu.cn (C.Y.); 2 Dalian University of Science and Technology, Dalian 116052, China

**Keywords:** underwater wireless sensor networks, opportunistic routing, Q-learning technique, depth-based routing, energy-efficient

## Abstract

Underwater Wireless Sensor Networks (UWSNs) have aroused increasing interest of many researchers in industry, military, commerce and academe recently. Due to the harsh underwater environment, energy efficiency is a significant theme should be considered for routing in UWSNs. Underwater positioning is also a particularly tricky task since the high attenuation of radio-frequency signals in UWSNs. In this paper, we propose an energy-efficient depth-based opportunistic routing algorithm with Q-learning (EDORQ) for UWSNs to guarantee the energy-saving and reliable data transmission. It combines the respective advantages of Q-learning technique and opportunistic routing (OR) algorithm without the full-dimensional location information to improve the network performance in terms of energy consumption, average network overhead and packet delivery ratio. In EDORQ, the void detection factor, residual energy and depth information of candidate nodes are jointly considered when defining the Q-value function, which contributes to proactively detecting void nodes in advance, meanwhile, reducing energy consumption. In addition, a simple and scalable void node recovery mode is proposed for the selection of candidate set so as to rescue packets that are stuck in void nodes unfortunately. Furthermore, we design a novel method to set the holding time for the schedule of packet forwarding base on Q-value so as to alleviate the packet collision and redundant transmission. We conduct extensive simulations to evaluate the performance of our proposed algorithm and compare it with other three routing algorithms on Aqua-sim platform (NS2). The results show that the proposed algorithm significantly improve the performance in terms of energy efficiency, packet delivery ratio and average network overhead without sacrificing too much average packet delay.

## 1. Introduction

With the great application prospects in marine environmental protection, underwater exploration, marine disaster monitoring, offshore operations and marine military activities, Underwater Wireless Sensor Networks (UWSNs) have drawn a great attention from governments, industry and academia over the course of the past few years [1,2]. Routing is a non-trivial task in UWSNs that can ensure fast and reliable transmission of data packets, which becomes one of the most valuable research hotspots recently.

Designing an efficient and reliable routing algorithm is a fundamental but essential research topic in UWSNs. However, it is much more difficult to communicate in UWSNs than in terrestrial sensor networks due to the unique characteristics of UWSNs [3,4]. The sensor nodes in UWSNs are usually powered by batteries, which is challenging to replace or recharge them because of the harsh and violent underwater environment. Therefore, improving the energy efficiency is one of the most crucial concerns that should be considered in the design of routing algorithms for UWSNs. In addition, radio signals attenuate rapidly in underwater environment. Larger antennas and higher transmission power are required to propagate at longer distance with extra low frequencies [3,4,5,6]. Besides, optical signals are affected by the factors of water clarity, scattering and precision in UWSNs [6]. Both radio and optical signals cannot meet the requirement of long-distance underwater communication. Thus, acoustic signals are usually adopted in UWSNs to communicate. However, compared with the radio-frequency channel, the bandwidth of an acoustic channel is lower (up to 20kbps) and the propagation delay is longer (approximately 1500 m/s) [7]. Meanwhile, it may also be affected by some unfavorable factors such as path loss, noise and Doppler spreading, which can cause extremely high packet loss ratio and total energy consumption [2,6]. What is more, the network topology changes frequently in UWSNs since the sensor nodes move passively with the water currents [8,9]. To summarize, the aforementioned inherent features of UWSNs make it difficult to design efficient and appropriate routing algorithms. Due to all the above differences between the terrestrial sensor networks and UWSNs, traditional routing algorithms for terrestrial-based wireless sensor networks are not feasible for UWSNs [10]. Hence specific routing algorithms must be designed aiming at the intrinsic properties in UWSNs. 

Underwater opportunistic routing (OR) algorithms have been proposed as a promising paradigm to mitigate these drawbacks and improve the performance of network in an underwater environment [11]. Unlike conventional routing algorithms in which packets are transmitted along the pre-defined route and a specific neighbor node is selected as the next-hop forwarder, OR algorithms make good use of the broadcast characteristic of wireless channels to select a set of next-hop forwarder candidates [12]. These candidates provide better alternative paths to the destination so that it can adapt to the dynamic underwater topology effectively and reduce the negative effect of weak links in UWSNs. It can also increase the communication reliability and network throughput by taking advantage of multiple potential relay nodes. However, there are lots of challenges in order to obtain a better performance in UWSNs with the use of OR algorithms [13,14,15]. A large number of redundant copies can be generated due to the usage of multiple potential candidates during forwarding process, resulting in unnecessary energy losses. Furthermore, the frequently forwarding of some high priority nodes leads to energy exhausted early, which shortens the network lifetime and causes the routing void that occurs whenever the current node has no neighbor closer to the water surface than itself for packet forwarding to the destination. A node is a void node if none of its neighbor nodes makes a positive progress for packet transmission towards the sink on the water surface [16]. Some void-handling approaches are required in OR algorithms to achieve a recovery when the packet reaches a void node [16]. A comprehensive review for the currently available void-handling approaches in UWSNs is presented in Reference [16], in which a classification and a comparison of the current void-handling techniques and the main features for designing efficient void-handling approaches have also been investigated.

In general, OR algorithms are composed of two main procedures, that is, candidate set selection and candidate set coordination. The candidate set selection procedure is for the purpose of selecting a suitable set of neighboring nodes as potential candidates, which can be further classified into three groups: sender-side-based, receiver-side-based and hybrid approaches [17,18]. The candidate set coordination process is responsible to choose relay nodes from candidates and suppress redundant packet transmissions of low-priority nodes. The underwater OR algorithms can be roughly divided into two main categories: localization-based and localization-free. The significant difference between them is whether or not the awareness of location by the sensor nodes [19]. A focused beam routing (FBR) in Reference [20] is a localization-based OR algorithm to address energy consumption. It applies the exchanging of RTS and CTS to select forwarders within a cone formed from the source to the destination. It can improve the energy efficiency but it has a low packet delivery ratio in sparse conditions. 

Due to the accurate three-dimensional information is hard to achieve in an underwater environment, localization-based routing algorithms are difficult to be widely applied. While localization-free algorithms pick out packet forwarders only according to the depth information of the nodes which can be obtained easily [21]. A depth-based routing (DBR) is proposed for UWSNs in Reference [22], which requires water pressure (depth) as a forwarder’s selection metric. It achieves great performance improvements in packet delivery ratio and end-to-end delay. However, it has high load on the nodes closer to the water surface. 

As a classic learning method that interacts with the environment, reinforcement learning is one of the most critical contents in the field of artificial intelligence and machine learning [23]. It does not require agents to grasp the environment model in advance, nor does it require supervision and guidance from supervisors. The Q-learning algorithm proposed in Reference [24] is one of the most representative and widely used reinforcement learning algorithms. In the Q-learning algorithm, agents learn continuously to adapt to the environment better according to their received rewards or penalties. Thus, it has great adaptability and flexibility in practical applications [25]. In recent years, several routing algorithms [26,27,28,29] based on Q-learning technique are implemented in UWSNs. The QELAR algorithm [26] applies machine-learning technique in the distributed underwater routing for the purpose of energy conservation and lifetime extension. But lots of control packets are used to exchange the related information, resulting in unnecessary overhead. The authors propose a QDAR algorithm in Reference [27] for underwater networks, which aiming to extend the lifetime of UWSNs with the aid of Q-learning algorithm. However, the designated routing path needs to be constructed before issuing the data packet, which leading to high end-to-end delay due to the low acoustic velocity. Moreover, the frequent construction of routing paths brings about plenty of additional overheads and energy consumption. With AOA and RSS positioning mechanism, a reinforcement learning based data forwarding algorithm is proposed in Reference [28] to cope with the passive mobility of nodes in underwater routing. By adopting the Q-learning to build the mobile model of sensor nodes, it achieves a good performance in terms of energy consumption but has a low packet delivery ratio. A localization-based routing algorithm based on Q-learning with additional kinematics and sweeping features (QKS) is proposed in Reference [29]. It has great adaptability to the dynamic topology by estimating the node velocity and position with the help of Q-learning. However, it is very difficult to realize underwater location.

In summary, the depth-based opportunistic routing tackles the harsh underwater environment well but it only focuses on the local optimal selection by employing greedy approaches. Although the aforementioned routing algorithms based on reinforcement learning for UWSNs can improve the network performance in some aspects by observing and learning the environment, some of them cannot control the routing overhead well when exchange the related information about Q-values and the others require position information, in which it is a tricky to achieve accurate underwater location. Meanwhile, as far as we know, the Q-learning technique has not been introduced to underwater depth-based OR algorithms basically. Motivated by the above considerations, in this paper, we propose an energy-efficient depth-based OR algorithm with Q-learning (EDORQ) to further reduce the energy consumption and improve the robustness for UWSNs. Instead of depending on full-dimensional position coordinates for packet delivery, the EDORQ needs only local depth information which can be easily obtained via an inexpensive pressure sensor. Our main contributions can be summarized as follows.

- We introduce the Q-learning technique into the underwater OR algorithm, so as to fully utilize their respective advantages. On the one hand, it takes full advantage of the broadcast nature of wireless medium in OR for reliable data packet delivery. On the other hand, global optimization can be achieved through single step Q-value iteration with the aid of Q-learning technique, which overcomes the shortcomings of greedy strategy and local optimization in traditional underwater OR algorithms.
- In our proposal, the void detection factor, residual energy and depth information of nodes are jointly considered in the reward function of Q-learning, which contributes to proactively detecting and bypassing void nodes in advance as well as avoids excessive energy consumption for the nodes locating in hot regions.
- We design a simple and scalable void node recovery mode in the candidate set selection phase through a packet backward retransmission manner to recover packets that encounter void nodes unfortunately. Instead of the network topology information, the void recovery mode needs only local depth to go around void nodes, which can further improve the packet delivery ratio, especially in a sparse network.
- We exploit a timer-based candidate set coordination approach to schedule packet forwarding, where a novel method to set the holding time is designed on the basis of Q-value, which helps to reduce the packet collisions and redundant transmissions. Besides, the Q-value is shared in only one hop neighbor, which is beneficial to further decrease overhead and energy cost.

The remainder of this paper is organized as follows. The related works are reviewed comprehensively in Section 2. Section 3 illustrates the underwater architecture and Q-learning model in UWSNs. The proposed algorithm is described in detail in Section 4. We report the simulation results and analysis in Section 5. Section 6 concludes this paper and refers to future works. 

## 2. Related Work

As a special wireless sensor network, the underwater routing algorithm is required to be deliberately tailored to fit the unique characteristics, which poses great challenges to the design of underwater routing algorithms for UWSNs [30]. In this section, we review the related research works on this topic.

Xie et al. propose a vector-based forwarding (VBF) routing algorithm for UWSNs in Reference [31]. A virtual “pipe” from the source to the destination is predefined for the selection of forwarder nodes. The nodes only located in the routing “pipe” can be selected as the qualified candidates. It avoids excessive redundant transmissions since the low priority nodes are suppressed. However, frequent usage of sensor nodes in “pipe” leads to early death of these nodes. As a result, it will disconnect the routing links and cause the energy distribution unbalanced. A hop-by-hop vector-based forwarding algorithm (HH-VBF) is proposed in Reference [32] to mitigate the drawbacks of VBF. HH-VBF creates a dynamic virtual routing “pipe” in each hop instead of a fixed vector from the source to the sink. It is less sensitive to “pipe” radius threshold and has a higher opportunity to deliver the packet to the destination in the sparse network. Simulation results show that HH-VBF has better performance of packet delivery ratio and more robustness than VBF. However, more energy is consumed in a dense network due to the dynamic change of the routing “pipe.” Furthermore, both VBF and HH-VBF are localization-based OR algorithms, which require sensor nodes to know their own full-dimensional position coordinates information. However, as mentioned before, it is not an easy task for underwater sensor nodes to obtain their location information, which limits the usage of VBF and HH-VBF in practical applications. 

In Reference [22], the first localization-free routing algorithm named as depth-based routing (DBR) is proposed. As a representative opportunistic routing algorithm, DBR adopts the holding time to schedule packet forwarding. During the holding time, the sensor nodes with the highest priority will suppress the packet forwarding of the other nodes with the lower priority. A fixed depth threshold mechanism is introduced to reduce the number of redundant packets. It requires that the depth difference between the previous hop node and candidate node should larger than a fixed depth threshold. The mechanism decreases the number of redundant packets to a certain extent in a dense network. However, the defect of this mechanism is that the depth threshold is set to a fixed value even if the underwater nodes are not deployed uniformly. If the value is too large, the packet delivery ratio will be very small in sparse networks. Contrariwise, the number of redundant packets will increase in dense networks if the value is too small. It cannot adapt well to the changing of the senor density.

Aiming at the problem of fixed depth threshold in DBR, the DEADS algorithm proposed in Reference [33] employs an optimized depth threshold mechanism. Three optimal depth threshold values are defined in advance to reduce the scope of candidate set. The source node determines which optimal depth threshold value should be taken on the basis of the number of dead nodes in the network. The algorithm can overcome the defect that the fixed depth threshold only has one value in DBR to a certain extent. However, there are only three optimal values in the dynamic network cannot adapt to the changing density of sensor nodes well. Furthermore, it needs the whole network information to select the depth threshold that greatly increases the overhead. 

**Figure 1:**
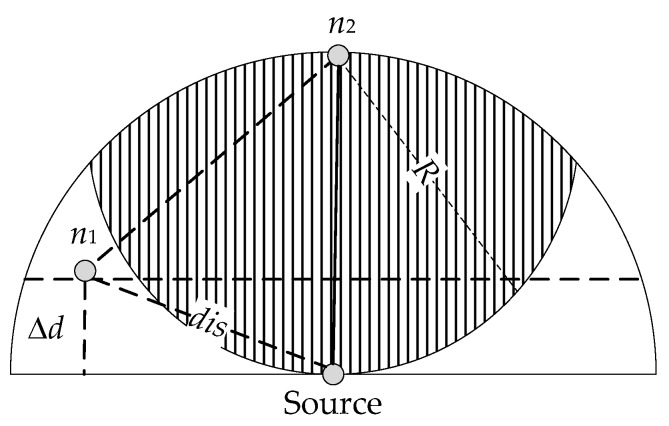
Forwarding zone of energy-efficient depth-based routing algorithm (EE-DBR) [34].

Diao et al. propose an energy-efficient depth-based routing algorithm (EE-DBR) in Reference [34], in which an underwater time of arrival (ToA) ranging technique [35] is introduced to reduce multipath redundancy forwarding. Instead of using the depth threshold mechanism, EE-DBR removes the redundant nodes in the blind zone (nonshaded area in Figure 1) to select the next-hop forwarder candidates. The calculation of blind zone is given as Equation (1).

(1) $$(dis^{2}-\Delta d^{2})+{\left(R-\Delta d\right)}^{2}>R^{2},$$

where *dis* is the minimal distance between node *n_1_* and source node, which can be obtained by ToA technique. Δd is the depth difference of two nodes and *R* is the maximum transmission range of one hop. EE-DBR effectively reduces redundancy energy waste in the blind zone compared with DBR. However, there are still a large number of redundant copies in the non-blind zone in a dense network. What is more, it is challenging to guarantee the synchronization of time when using ToA technique in UWSNs. 

In Reference [36], Ullah et al. propose two routing mechanisms of effective energy and reliable delivery (EERD) and cooperative effective energy and reliable delivery (CoEERD) for UWSNs to improve the energy efficiency and reliability of packet transmissions. EERD is a single path routing mechanism, in which a certain weight function based on residual energy, bit error rate and shortest distance is designed to prolong the network lifetime and reduce the energy consumption and delay. In order to ensure packet reliability in harsh underwater conditions, the CoEERD, a multiple path routing mechanism, improves the EERD by adding the cooperation of a single relay node between a source-destination pair. However, it increases the energy usage and average delay compared with EERD due to the cooperation of nodes.

Energy balanced localization-free cooperative noise-aware routing for UWSNs is investigated in Reference [37] and two protocols are proposed. One is depth and noise-aware routing (DNAR), which is designed to reduce the energy consumption by combining the extent of link noise with the depth of a node. But only a single link is utilized to send data, which is vulnerable to the harshness of channel. The other is cooperative DNAR (Co-DNAR) algorithm proposed to overcome the weaknesses of DNAR. In Co-DNAR, the source-relay-destination triplets in information advancement are applied to reduce the probability of information corruption while with a high data traffic on the relay and the destination nodes.

The above-mentioned OR algorithms can adapt to the UWSNs well but they do not have void-handling techniques to deal with the routing void problem. In Reference [38], the authors propose a void-aware pressure routing (VAPR) algorithm to enhance the performance of opportunistic directional forwarding. The sequence number, hop count and depth information are used to build a directional trail to the closest sonobuoy, which does not need additional recovery path maintenance. Due to whole network beacon propagation being involved to share the control information, undoubtedly it increases the network overhead. 

Lee et al. propose a hydraulic-pressure-based anycast routing (HydroCast) algorithm in Reference [39] to address the challenges of ocean current and limited resources in UWSNs. HydroCast selects a cluster of candidates with the maximum progress and limited hidden terminals by exploiting the hydraulic pressure level of sensor nodes. A dead-end recovery method is proposed to improve the performance of simple 3-D flooding manner. However, as a sender-side-based OR algorithm, it updates the neighbors’ information frequently for the selection of next-hop forwarder, which greatly increases the energy consumption. 

An opportunistic void avoidance routing (OVAR) is proposed in Reference [40] to handle the void problem and improve the energy efficiency in UWSNs. In OVAR, the adjacency graph is constructed at each hop, which does not require global topology information to impose less overhead. The packet delivery probability and packet advancement are utilized to select the relay nodes, which increases the throughput and reliability in a sparse network. However, the nodes close to the sinks are frequently involved in packet forwarding, leading to the unbalanced energy consumption of nodes [4].

Hu et al. [26] propose a machine-learning-based energy-efficient and lifetime-aware routing (QELAR) algorithm for UWSNs. The Q-learning technique is applied in QELAR so as to learn and adapt to the dynamic underwater environment efficiently. The residual energy of each node is considered into the definition of reward function to prolong the lifetime of network. However, it adopts a large number of “metadata” packets to exchange the information, introducing plenty of overhead and collisions unavoidably. The more sensor nodes in the network, the higher energy will be consumed. Thus it is unsuitable for dense networks with a great quantity of nodes. 

A Q-learning-based delay-aware routing algorithm (QDBA) is proposed in Reference [27] to extend the network lifetime and reduce end-to-end delay. It defines an action-utility function with residual energy-related cost and delay-related cost for routing decisions. The defined “DATA_READY” and “INTEREST” control packets are applied to construct the routing path between sources and sink nodes. However, the designated paths may be easily broken before data packets are sent due to the dynamic topology in UWSNs. Besides, the frequent construction of routing paths results in lots of extra overheads and energy consumption.

Chang et al. propose a reinforcement learning-based data forwarding algorithm in UWSNs with passive mobility [28]. The performance of both delay and energy consumption are improved in a specific dynamic scene. AOA and RSS positioning mechanism are used in the phase of orientation determination but the precision is difficult to be guaranteed in UWSNs. What is more, it sacrifices the packet delivery ratio greatly. Thus it is inappropriate for the delivery ratio sensitive scenarios.

In Reference [29], the authors propose an underwater routing algorithm based on Q-learning with additional kinematic and sweeping characteristics (QKS). The transmission probabilities in Q-value are modeled based on the position and velocity of nodes in order to handle the nodes’ high mobility in underwater environment. However, the underwater positioning is a trouble due to the high attenuation of RF and the velocities of nodes in QKS are estimated by Kalman filter, which is also inaccurate underwater.

In conclusion, the aforementioned underwater routing based on reinforcement learning technique enhances the network performance in many aspects, through observing and learning the environment. However, the routing overhead is not controlled well when share the related information of Q-values. Besides, it is few and far between for the existing routing algorithms to concentrate on the combination of depth-based OR routing and Q-learning technique in UWSNs, which can make full use of their advantages, respectively. In this paper, we propose an energy-efficient depth-based OR algorithm with Q-learning technique (EDORQ) for UWSNs to further reduce the total energy consumption and improve packet delivery ratio. Our algorithm is different from those above as follows. First, the Q-learning technique is utilized in OR algorithm to learn the environment and adapt to the dynamic underwater topology. Second, we take the void detection factor, residual energy and depth difference information of sensor nodes into account in the construct of Q-value for the void detection and avoidance, which is beneficial to balance the energy distribution, reduce energy consumption and improve the packet delivery ratio. Next, a simple void recovery mode is designed to select next hop candidate nodes, which further minimizes the unnecessary energy waste and improves the packet delivery ratio in a sparse network. Moreover, the timer-based candidate set coordination approach based on Q-value is applied to reduce the collisions of the packets while imposing minimal overhead. Finally, extensive experiments are conducted on the popular Network Simulator Version 2 (NS2) [41] platform. The simulation results demonstrate that our proposal can significantly improve the performance in terms of energy efficiency, packet delivery ratio and average network overhead.

## 3. System Model 

### 3.1. Network Architecture

**Figure 2:**
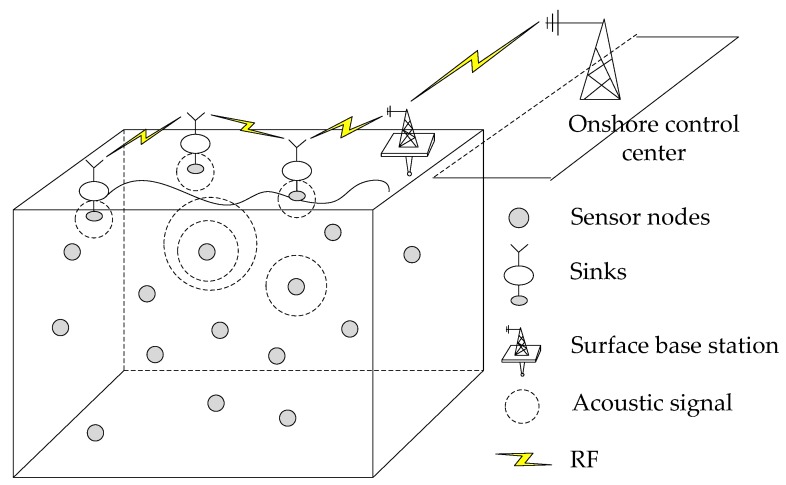
Underwater sensor network architecture.

The UWSN architecture considered in this paper is depicted in Figure 2, which is composed of underwater sensor nodes, sinks, underwater acoustic channels and water surface base station. A large number of wireless sensor nodes with acoustic modems are randomly deployed underwater with different depths, operating to collect oceanographic data. Multiple sinks are randomly placed on the water surface and equipped with both radio-frequency and acoustic modems. On the one hand, sinks adopt acoustic signals to receive data packets from underwater sensor nodes. On the other hand, the radio modem is employed by sinks to communicate with the surface base station. 

The base station transmits the collected data to the onshore control center for offline information processing. Suppose that with the aid of an inexpensive depth sensor, each sensor node knows its own depth [22], that is, the minimum distance from itself to the water surface. What we discussed is the routing process that data packets are transmitted from underwater sensors to sinks.

### 3.2. Q-Learning Model 

#### 3.2.1. Markov Decision Process (MDP) Model

The MDP is the optimal decision process of stochastic dynamic system and also the theoretical basis of reinforcement learning. The routing decision process of the entire UWSN can be regarded as a reinforcement learning system. And the routing problem can be formulated as a discrete MDP [23]. The mathematical framework of MDP consists of a tuple of <*S*, *A*, *P*, *R*>, where *S*, *A*, *P*, *R* are the set of discrete states, actions, state transition probabilities and rewards, respectively. The related definitions in our routing scenario are explained as follows.

**Agents:** Each underwater sensor node is regarded as an independent agent. Sensor nodes distributively learn from the underwater environment to transmit packets, which are described by a finite set of *N* = {*n*_1_*,n*_2_*,……n_j_……n_m_*}, where *n_j_* is the *j*th sensor node and *m* is the total number of sensors. 

**States:***S* = {*s*_1_*,s*_2_*,…s_j_…s_m_*} is defined as the states set of the network, where the state *s_j_* represents that the data packet reaches to the node *n_j_*. If a packet is forwarded from node *n_i_* to node *n_j_*, then the state of node transfers from *s_i_* to *s_j_*.

**Actions:***A* = {*a*_1_*,a*_2_*,…a_j_*} denotes the set of exploration actions, where the action *a_j_* represents the node *n_j_* is selected as the next hop forwarder successfully. 

**Reward:**$R_{s_{i}s_{j}}^{a_{j}}$ represents an immediate reward (positive or negative) when an agent takes action *a_j_* to make a state transfer from *s_i_* to *s_j_*. The factors to be considered in the construction of the reward function may include energy consumption, network lifetime, average end-to-end delay and so forth, which depends on the specific task of the scenario. 

**Probability Transfer Function:**$P_{s_{i}s_{j}}^{a_{j}}$ indicates the transition probability that node *n_i_* takes the action *a_j_* from state *s_i_* to state *s_j_* successfully, while the failure transition probability is defined as $P_{s_{i}s_{i}}^{a_{j}}=1-P_{s_{i}s_{j}}^{a_{j}}$ [26]. In our model, the transition probability can be estimated at run time based on the success or failure history of node’s forwarding action.

#### 3.2.2. The Basic Q-Learning Technique

**Figure 3:**
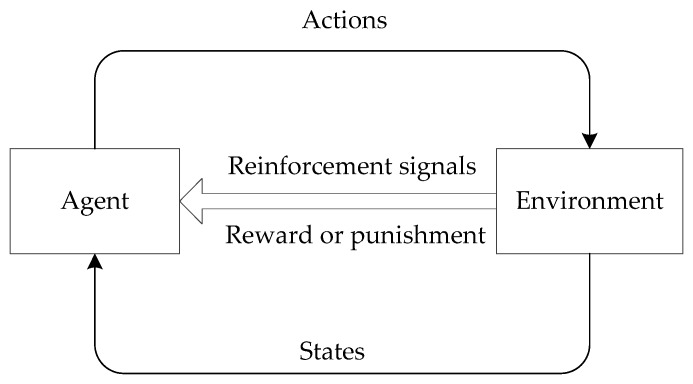
Schematic of reinforcement learning.

Reinforcement learning adopts a "trial-and-error" scheme to interact with the environment, committing to find the optimal behavioral policy to maximize the cumulative rewards [42]. Figure 3 depicts the basic pattern of reinforcement learning technique. The agent perceives the current state and the corresponding reinforcement signals (reward or punishment values) from the environment and then to perform an action. The quality of the action selection affects the next state and rewards. If an action makes the environment generate positive rewards, the trend of agent to select this action will be strengthened. With the passage of time, the agent will learn an optimal behavioral policy to get a higher reward.

Q-learning is a value-based reinforcement learning technique, which determines an optimal policy to obtain a higher reward in a step-by-step iteration manner. It can evaluate the performance of a given action at a particular state with the aid of the Q-value (action-value), *Q*(*s_i_*,*a_j_*), which denotes the expected discounted reward for taking an action *a_j_* at the state *s_i_* [24]. The Q-value function satisfies the Equation (2):

(2) $$Q(s_{i},a_{j})=r_{i}(a_{j})+\gamma \sum_{s_{j}\in S}^{}P_{s_{i}s_{j}}^{a_{j}}\underset{a}{\max}Q(s_{j},a),$$

where γ(0≤γ<1) is the discount factor, which is used to determine the importance of future rewards. When γ is small, the agent will pay more attention to the immediate rewards. Conversely, the agent will pay more attention to the future rewards. Typically, to balance the direct and future reward, the value of γ is within (0.5, 0.99) [26]. *r_i_(a_j_)* is the direct reward function, which is critical to Q-learning as it determines the behavior and performance of action *a_j_* at the state *s_i_*, which can be defined as follows:

(3) $$r_{i}(a_{j})=\sum_{s_{j}\in S}^{}P_{s_{i}s_{j}}^{a_{j}}R_{s_{i}s_{j}}^{a_{j}}.$$

According to the Bellman’s principle of optimality [43] in dynamic programming, once the maximum Q-value function is found, the optimal policy can be obtained. The optimal action a_i_* at state *s_i_* can be acquired as follows.

(4) $$a_{i}{}^{*}=\arg\underset{a_{i}\in A(s_{i})}{\max Q(s_{i},a_{i})},$$

where *A*(*s_i_*) is the set of actions that *a_i_* may be chosen at state *s_i_*. A greedy Q-learning algorithm always chooses the action with the highest Q-value, which contributes to the packets being forwarded from source to sinks via the best path.

## 4. EDORQ Algorithm

### 4.1. Overview of EDORQ

**Figure 4:**
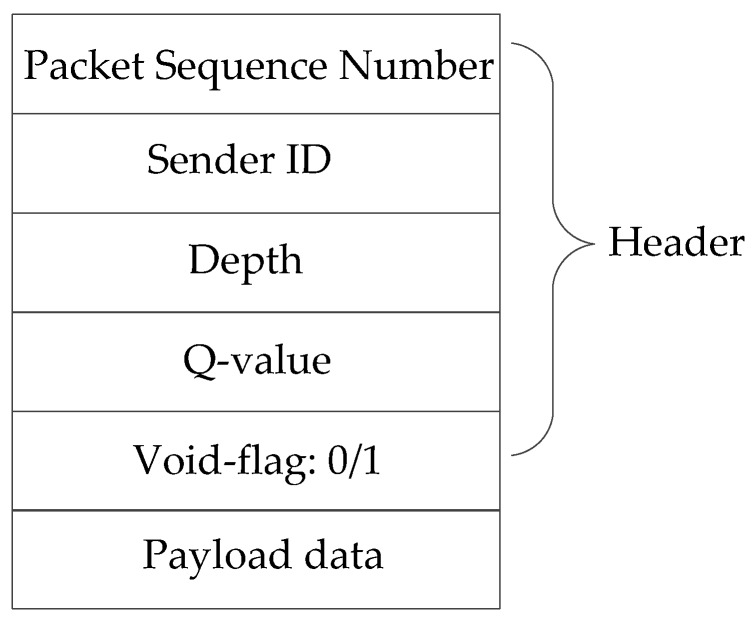
The format of data packets.

The routing process of our proposed algorithm EDORQ includes two phases—candidate set selection phase and candidate set coordination phase. The purpose of the first phase is to select a subset of neighbor nodes as candidates to continue forwarding the packet toward the destination. The general candidate set selection method can be divided into two different categories: sender-side-based and receiver-side-based [18]. In the sender-side-based method, the unique ID of candidate nodes is embedded into the data packet by sender to determine the next forwarders. Therefore, lots of control packets are exchanged between nodes to acquire the network topological information. To avoid huge overhead of the network, our proposal employs receiver-side-based candidate set selection method without extra control packets. As a simple and scalable candidate set selection method, it requires that packet receivers decide whether or not to be qualified candidates by themselves. Because of this, energy conservation and channel utilization improvement can be achieved. In EDORQ, the data packet is composed of two parts: packet header and payload data. Its format is illustrated in Figure 4, where the packet header consists of five fields, as follows.

Packet Sequence Number: The unique sequence number of the packet.

Sender ID: Node ID of the node sending the data packet.

Depth: The depth information of the current node.

Q-value: The Q value of the current node.

Void-flag: A bit information of “0” or “1,” identifying whether or not the current forwarder of the packet is a void node. If the node is a void node, the void-flag is filled with “1”; otherwise the void-flag is filled with “0.”

In the second phase, the candidate nodes will collaborate with each other to decide the forwarding orders with an attempt to suppress the redundant forwarding according to their priorities. To achieve that, the common technique of a timer-based mechanism [17] is applied in EDORQ. However, in order to further minimize the energy consumption and raise the dynamic adaptability, the Q-learning technique is adopted in our proposal to design the holding time mechanism, which is further elaborated in Section 4.3. The different holding time are assigned to each neighbor node in the candidate set based on Q-value. The potential candidate node with the highest Q-value is first selected as the best forwarder.

### 4.2. Void-Detection Based Candidate Set Selection

So as to show the process for candidate set selection clearly, we assume that an intermediate node *n_i_* is the current forwarder aiming to determine the candidate set to continue packet transmission to sinks. *CS(n_i_)* is considered as the candidate set of node *n_i_*. The number of nodes in *CS(n_i_)* has a significant influence on the routing performance. If the number is too small, the packet delivery ratio will be low; otherwise, the energy consumption will be increased. The depth and void-flag information embedded in the data packet header are utilized as the metrics to select the candidate set. 

**Figure 5:**
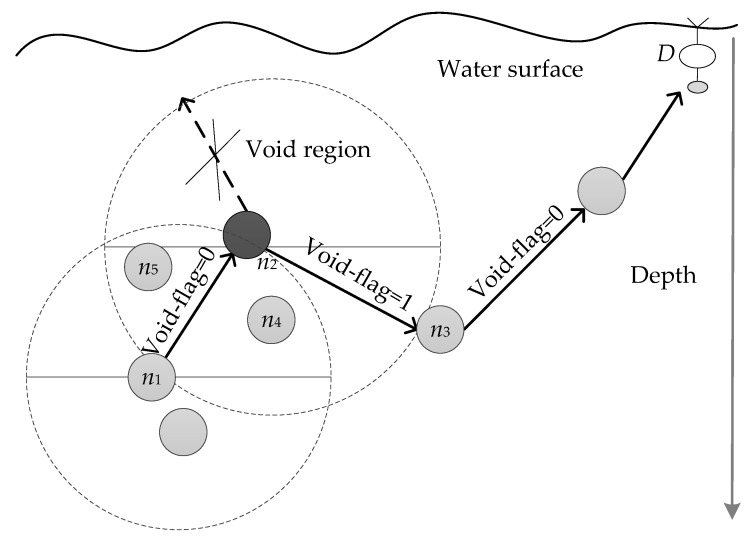
The void recovery mode.

We divide the candidate set selection procedure into two modes of greedy mode and void recovery mode. The greedy mode is first applied to select candidate forwarders, while the void recovery mode is actuated whenever the routing void is encountered. *n_i_* first broadcasts the data packet to its one hop neighbor. After receiving the data packet, each neighbor node extracts the depth *d_i_* and void-flag information of *n_i_* from the packet header and then compares *d_i_* with its own depth. The greedy mode contributes to finding a set of candidate nodes closer to the water surface, which can ensure that the data packets are transmitted upwards to the sinks quickly. To this end, the void-flag field in the header of packet is filled with “0,” indicating that only the nodes with a smaller depth than the current forwarder can be selected as an eligible candidate. The neighbors with a bigger depth than the current forwarder are not desirable nodes as next forwarders, only discarding the data packet simply. However, one major defect should be properly addressed in the greedy mode is that the data packet may get stuck in a void node, that is, there is no qualified candidate node in its lower depth region. As shown in Figure 5, node *n*_2_ is the next forwarder of *n*_1_. After receiving the packet from *n*_1_, node *n*_2_ first updates the void-flag in packet header with “0” and then sends out it with the greedy mode. If it does not hear that the packet is successfully forwarded during a period of time, then the void recovery mode will be triggered. Subsequently, node *n*_2_ will retransmit the data packet in a void recovery mode, where the void-flag filed is filled with “1,” allowing those neighbor nodes with greater depth to be selected as candidate nodes. To suppress duplicate packets, similar to the DBR [22], ideally a node forwards the packet with the same ID only once in a certain time interval. Accordingly, nodes *n*_3_, *n*_4_ and *n*_5_ form the new candidate set of node *n*_2_. Node *n*_3_ is the optimal next forwarder of *n*_2_ and then it will continue the packet transmission in a greedy mode toward to the sink *D* until the void node occurs. 

The phase of void-detection based candidate set selection is conducted in Algorithm 1 and Algorithm 2 describes the switching mechanism of greedy mode and recovery mode.

**Algorithm 1** Candidate Set SelectionInput: the packet *p* broadcasted by *n_i_* Output: *CS*(*n_i_*) //the candidate set of *n_i_* for packet forwarding1:*CS*(*n_i_*)=∅2:**for** each neighbor node of *n_i_* denoted by *n_j_* **do**3: **if** it has forwarded the packet *p* **then**4:  drop *p*5: **else**6:  extract the depth *d_i_* and *n_i_*.void-flag from *p*7:  obtain its own depth *d_j_*8:  compute $\Delta d=d_{i}-d_{j}$9:  **if** (!*n_i_*.void-flag and Δd > 0) or (*n_i_*.void-flag) **then**10:   *CS*(*n_i_*) = *CS*(*n_i_*) ∪ {*n_j_*}11:   **end if**12: **end if**13:**end for**

**Algorithm 2** Forwarding Mode SwitchInput: the packet *p* received by node *n_i_* Output: greedy mode or void recovery mode1:node *n_i_* updates *p* with *d_i_*2:*n_i_*.void-flag=03:BroadcastFlag=True // indicate that whether or not node *n_i_* transmits *p*4:RecoveryFlag=True // indicate whether to activate void recovery mode or not5:**while** (BroadcastFlag) **do**6: *n_i_* broadcasts *p* and set a specific timer7:  **while** (timer is not expired) and (RecoveryFlag) **do**8:   overhear the channel9:   **if** *n_i_* overhears *p* being transmitted **then**10:    RecoveryFlag=False11:   **end if**12:  **end while**13:**if** (RecoveryFlag) and (!*n_i_*.void-flag) **then** //overhear no transmission of *p* within its     //timer and it is the first time to activate the void recovery mode14: set *n_i_*.void-flag to 115:**else**16: drop the packet *p*17: BroadcastFlag=False18:**end if**19:**end while**


### 4.3. Q-Learning Based Candidate Set Coordination

In this subsection, we elaborate on the data forwarding coordination process among candidate nodes based on the Q-value, which is the expected discounted reward for executing an action at a particular state. Some of the main symbols used in this paper are listed in Table 1.

Suppose that node *n_j_* is one of the candidate nodes of current forwarder *n_i_*. In order to make packets detect and bypass the void node in advance as much as possible, meanwhile, minimize energy consumption, we define the immediate reward function $R_{s_{i}s_{j}}^{a_{j}}$ for taking action *a_j_* at state *s_i_* via an exponential function, as follows:

(5) $$R_{s_{i}s_{j}}^{a_{j}}={\left(2+\alpha \rho_{j}\right)}^{\delta_{ij}\cdot [\beta E_{j}+(1-\beta )D_{ij}]},$$

(6) $$s.t.,\delta_{ij}=\{\begin{array}{cc} 1, & \mathrm{if}\;n_{i}\;\mathrm{forwards}\;a\;\mathrm{packet}\;\mathrm{to}\;n_{j}\;\mathrm{successfully}(s_{j}\neq s_{i})\\ -1, & \mathrm{otherwise}(s_{j}=s_{i}) \end{array},$$

where $\rho_{j}\geq 0$ is designated as a void detection factor, which represents the number of forwarding packets from neighbors with lower depth overheard by node *n_j_* in a period of time. The factor is approximately proportional to the number of potential qualified forwarders of *n_j_*, which is utilized to detect the void nodes in advance. The larger the $\rho_{j}$ is, the less likely for node *n_j_* to be a void node. We plus two to $\alpha \rho_{j}$ in order to guarantee the base of exponential function Equation (5) is always greater than 1. α(0<α≤1) is the adjustment coefficients of $\rho_{j}$. *E_j_* is the energy-related factor while *D_ij_* is the depth-related factor, which are defined as Equations (7) and (8), respectively. The parameter β∈(0,1) is the weight factor to balance the impact between *E_j_* and *D_ij_*.

(7) $$E_{j}=\frac{e_{j}^{r}}{e_{j}^{i}},$$

(8) $$D_{ij}=\frac{d_{i}-d_{j}}{R_{\max}},$$

where $e_{j}^{r}$ and $e_{j}^{i}$ are the residual energy and initial energy of node *n_j_*, respectively. Clearly, the more energy node *n_j_* remains, the more rewards it obtains. *d_i_* and *d_j_* are the depth of nodes *n_i_* and *n_j_*, respectively. *R*_max_ is the maximum communication range between two sensor nodes. In conventional depth-based routing algorithms [22], the nodes with smaller depth tend to be dead prematurely since they participate in forwarding data packets frequently. Unlike them, our proposed reward function considers both the depth difference and residual energy information of sensors at the same time, which can avoid excessive energy consumption for the nodes with smaller depth. From the Equations (5)-(6) we can see that, if the node *n_i_* forwards a packet to *n_j_* successfully, then the immediate reward function $R_{s_{i}s_{j}}^{a_{j}}$ is a monotone increasing function and its value is always more than 1. It indicates that any node with higher residual energy, lower depth or greater void detection factor has more rewards to be selected as a forwarder node. Otherwise,$R_{s_{i}s_{i}}^{a_{j}}$, the immediate reward function for the case that node *n_i_* fails to forward a packet to *n_j_*, is a monotone decreasing function in the range (0,1), which is considered as a penalty for packet delivery failure that is always less than the $R_{s_{i}s_{j}}^{a_{j}}$. For those undesirable candidate nodes, less residual energy, depth difference or void detection factor they have, more penalties they will pay. Therefore, the direct reward function for node *n_i_* taking an action *a_j_* is expressed as Equation (9).

(9) $$r_{i}(a_{j})=P_{s_{i}s_{j}}^{a_{j}}R_{s_{i}s_{j}}^{a_{j}}-P_{s_{i}s_{i}}^{a_{j}}R_{s_{i}s_{i}}^{a_{j}},$$

where the state transition probabilities $P_{s_{i}s_{i}}^{a_{j}}$ and $P_{s_{i}s_{j}}^{a_{j}}$ can be estimated by the probabilities of successful and failed packet forwarding, respectively.

The Q-value of *n_j_* can be updated according to Equation (10).

(10) $$Q(s_{i},a_{j})=r_{i}(a_{j})+\gamma \sum_{s_{j}\in S}^{}P_{s_{i}s_{j}}^{a_{j}}\underset{a}{\max}Q(s_{j},a).$$

Suppose that the Q-value of each node is 0 in the initial stage. If the next hop is one of the sinks, *n_i_* transmits the packet with the largest reward in its cache to sink directly.

If all the eligible candidate nodes of *n_i_* take part in forwarding the same packet, then it will result in larger overhead and higher energy consumption. To reduce this waste of energy, we assign a holding time for each candidate node with the principle that nodes with larger Q*-*value have higher priority and lower holding time. Each candidate node of the current forwarder obtains its Q-value through Equations (5)-(10) and then sets a respective holding time according to the Q-value. The holding time of node *n_j_* can be calculated as follows:

(11) $$T_{j}=[1-\frac{2}{\pi }\arctan Q(s_{i},a_{j})]T_{\max},$$

where Q(*s_i_*,*a_j_*) is the Q-value calculated by node *n_j_*, *T*_max_ is a pre-defined maximum holding time. In order to further reduce the number of redundant packets, the *T*_max_ should be long enough to be able to suppress the duplicate transmission of lower priority nodes before relaying the packet. Thus, *T*_max_ can be defined as Equation (12):

(12) $$T_{\max}=\frac{2R_{\max}}{v_{s}},$$

where *v_s_* is the propagation speed of sound in the water. 

**Figure 6:**
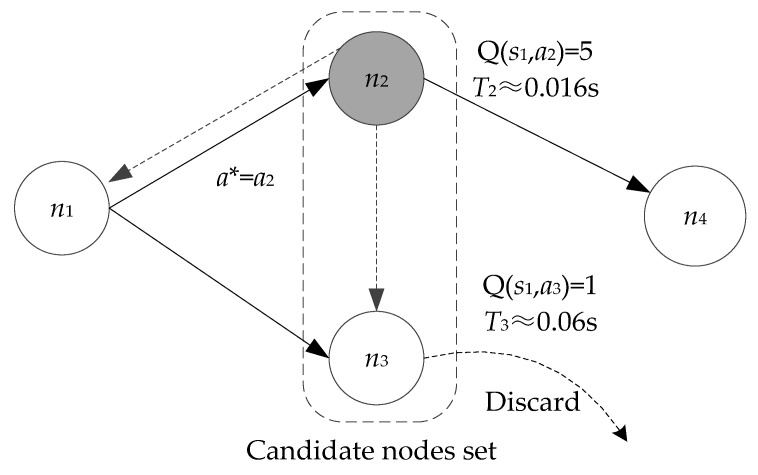
Timer-based candidate coordinate mechanism with Q-learning.

The candidate forwarder with a larger Q-value has a shorter holding time, which means that it is preferential to relay the data packet. If a candidate node with a lower priority overhears the same packet transmission of any node with a higher priority within its holding time, then it will discard this packet simply. A simple example is shown in Figure 6, nodes *n*_2_ and *n*_3_ are candidate forwarders of sender *n*_1_. It assumes that the respective Q-value of nodes *n*_2_ and *n*_3_ are 5 and 1. Upon receiving a data packet broadcasted by *n*_1_, nodes *n*_2_ and *n*_3_ start a timer, respectively. After calculating according to Equations (11)–(12), we know that the holding time of *n*_2_ is 0.016 s while *n*_3_ is 0.06 s. Consequently, the optimal action a* at state *s*_1_ is *a*_2_ and node *n*_2_ will first forwards the data packet when its timer expires. Node *n*_3_ will discard the packet simply when it overhears the same packet transmitted from node *n*_1_ during its holding time. This coordination approach has no additional control or ACK packets and is easy to implement. 

In our EDORQ algorithm, a sender node usually has several potential forwarding candidate nodes and each of them has opportunity to forward packets. If the sender overhears no transmission for the same packet from its candidate nodes after the maximum holding time, then the packet retransmission occurs. The sender node will select the new candidate set again in a void recovery mode to expand the range of candidate set. Algorithm 3 details the phase of Q-learning based candidate set coordination.

**Algorithm 3** Candidate Set Coordination Input: *CS* (*n_i_*) //candidate set of node *n_i_* Output: broadcast or drop the packet
1:**for** each node in *CS* (*n_i_*) denoted by *n_j_* **do**2: calculate its Q-value according to Equations (5)–(10)3: Set its holding time according to Equations (11)–(12)4: RelayFlag = True // indicates that whether or not a node overhears a packet within its     // holding time5: **while** (timer is not expired) and (RelayFlag) **do**6:  **if** overhear that packet *p* has been sent by any neighbor **then**7:   RelayFlag = False8:  **end if**9: **end while**10:**if** (RelayFlag) **then**11: broadcast the packet12:**else**13: drop the packet14:**end if**15:**end for**

### 4.4. Summary

In this subsection, we describe the data packet forwarding process from the perspective of an intermediate receiver sensor node. Upon receiving a data packet, the receiver node determines its eligibility to take part in the packet forwarding or not according to Algorithm 1. If it is not selected as a qualified candidate node, then only discards the data packet simply. Otherwise, it will implement the candidate set coordination phase by executing Algorithm 3. Firstly, it calculates the Q-value, which is an expect discount reward, on the bases of the void detection factor, depth difference and residual energy information and subsequently starts a timer with a holding time based on the Q-value to schedule packet forwarding. If the node does not hear that the same packet is forwarded from a higher priority node before itself holding time is expired, then it will relay the packet to continue the routing process in a greedy mode. After that, if it does not overhear the packet be transmitted after a period of time, the void recovery mode will be activated for the reselection of candidate set, in which the forwarding mode switch manner can be refer to the Algorithm 2.

The main strategy of our proposal is to select a qualified candidate set from one hop neighbor nodes and then to utilize the Q-learning technique for the determination of forwarder nodes with higher priority. In the candidate set selection phase, it takes a time complexity of *O* (*h*) at most to select the candidate nodes from neighbors, where *h* is the size of *CS* (*n_i_*). The time complexity of the second phase is similar to the algorithm proposed in Reference [28], which is O(*h*(*h*-1)). As a result, the complexity of our algorithm can be represented as *O* (*h^2^*).

## 5. Simulation Results and Analysis

### 5.1. Simulation Setup

In this paper, we evaluate the performance of the proposed EDORQ algorithm and compare it with VBF [31], DBR [22] and QELAR [26]. All simulations are implemented on the Network Simulator Version 2 (NS2) with an underwater sensor network simulation package (called Aqua-Sim) extension [41]. In our simulations, the source nodes are deployed at the bottom of the network randomly. Unless otherwise specified, four sinks are randomly deployed at the water surface. Each sink is equipped with both radio-frequency and acoustic modems. Once a packet arrives at any sink node successfully, it assumes that the packet reaches the destination. The other sensor nodes are randomly deployed in a 500 m × 500 m × 500 m 3-D underwater environment. They follow the random-walk mobility pattern [22]. Each of them moves to a new horizontal direction randomly with a random speed between 1 m/s and 3 m/s respectively, whereas the vertical movement is considered negligible [22]. Moreover, it adopts LinkQuest UWM1000 [44] as the acoustic modem with 10*k* bps transmission bit rate and a maximum transmission range of 100 m. These sensor nodes adopt the same propagation model, energy consumption model and initial energy. The discount factor γ is set as 0.9 [26], while the adjustment coefficients α is set as 0.5. To balance the depth with energy consumption, we set β is 0.5. In order to investigate the effect of node density on the performance of the four algorithms, we perform extensive simulations with different sizes of sensor nodes from 200 to 800, respectively. All of the simulation results are averaged over 20 runs for randomly-generated topologies with the 95% confidence interval. For each run, the simulation time is set to 800 s. The main simulation parameters are listed in Table 2. 

### 5.2. Simulation Metrics

The performances of the four algorithms are assessed by the following four metrics—total energy consumption, packet delivery ratio, average packet delay and average network overhead.

Total energy consumption (TEC) is the total amount of energy consumed by all underwater sensor nodes within the simulation duration, including the energy consumption of nodes in sending, receiving and idling mode.

Packet delivery ratio (PDR) is defined as the proportion of the number of packets successfully received by the sinks to the total number of packets sent by source nodes.

Average packet delay (APD) is the average time taken by a data packet transmission from the source to any of the sinks.

Average network overhead (ANO) is defined as the ratio of the total number of forwarding packets including data and control packets for all forwarder nodes to the number of data packets successfully delivered to any sink.

### 5.3. Simulation Results

#### 5.3.1. Performance Comparison

**Figure 7:**
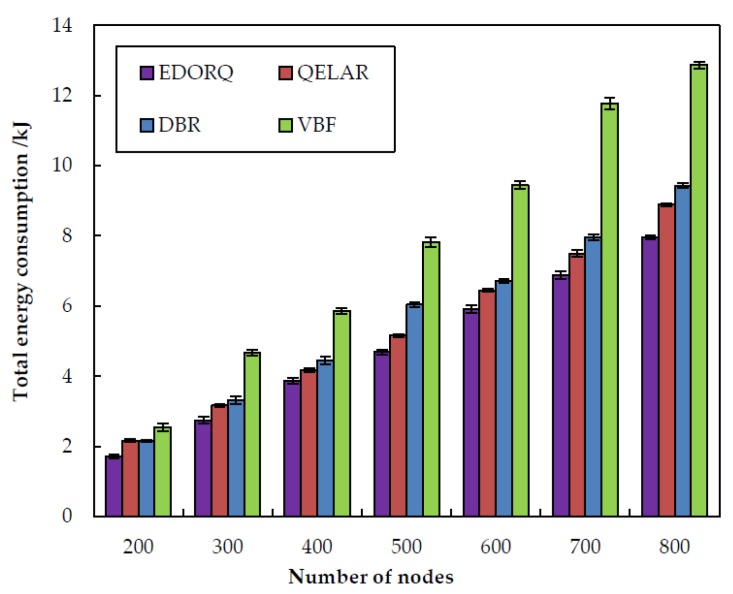
Total energy consumption.

Figure 7 compares the performance of VBF, DBR, QELAR and EDORQ in terms of total energy consumption for various numbers of nodes. We can observe that the total energy consumptions of the four algorithms increase with an increase of the sensor nodes. The reason is that when the density of nodes increases, the number of qualified forwarding nodes also increases, which causes more energy consumption in sending, receiving and even idling mode. It is notable that the EDORQ algorithm outperforms other counterparts, followed by QELAR, DBR and VBF in sequence no matter what the number of sensor nodes. This is attributed to that the use of void recovery mode and void detection factor reduces the chance to encounter a void node, which effectively reduces the energy consumption caused by routing void problem, while neither DBR nor VBF take it into account. What is more, its holding time based on Q-value of each candidate nodes prevents lots of redundant copies retransmissions that resulting in less energy consumption than the other three algorithms. In addition, in contrast to QELAR, the EDORQ algorithm does not require too many extra control packets to exchange network information, further avoiding excess energy consumption. QELAR consumes less energy since fewer nodes are involved in the packet forwarding process compared to DBR. The higher energy consumption result in VBF is mainly caused by a number of redundant transmissions. DBR adopts the fixed depth threshold mechanism to suppress the number of redundant packets, thus it is more efficient at energy saving than VBF. 

**Figure 8:**
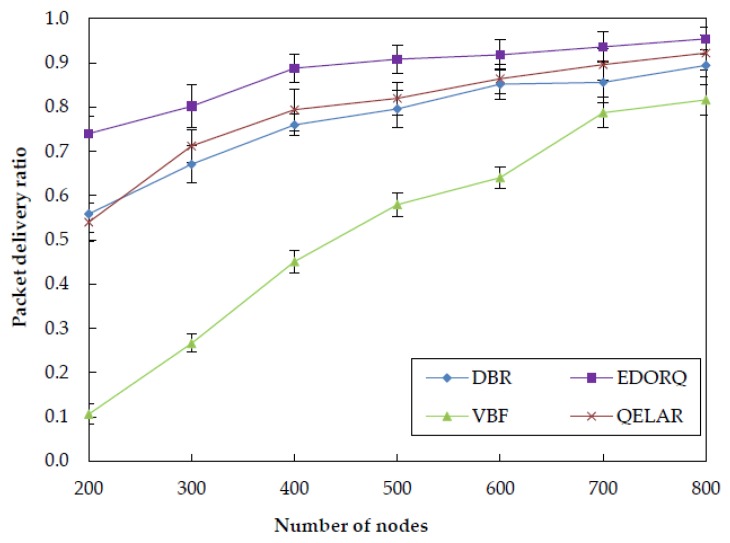
Packet delivery ratio.

Figure 8 depicts the performance of the four algorithms in term of packet delivery ratio with different number of nodes. It can be clearly seen that the packet delivery ratio is enhanced as the density being increased. This is because with the number of nodes increasing in the network, the more nodes have opportunity to be selected as suitable forwarder nodes, which leads to a higher packet deliver ratio. It can also be observed that when the number of nodes is greater than 400, DBR, QELAR and EDORQ can achieve a higher packet delivery ratio (more than 0.75) but this metric of VBF is always at a relatively low level (less than 0.5). The reason is that the packet delivery ratio of VBF is significantly influenced by the radius of routing “pipe” and the location precision underwater. The passive movement of sensor nodes in the networks affects the number of nodes in “pipe,” thereby reducing the packet delivery ratio of VBF. No matter what the number of sensor nodes, our algorithm has obvious advantages than the other three algorithms and the lower the node density is, the more obvious the advantage is. This is because the void detect factor in Q-value and the void recovery mode in candidate selection phase are used to improve the packet deliver ratio in EDORQ, which can achieve good performance especially in the sparse network.

**Figure 9:**
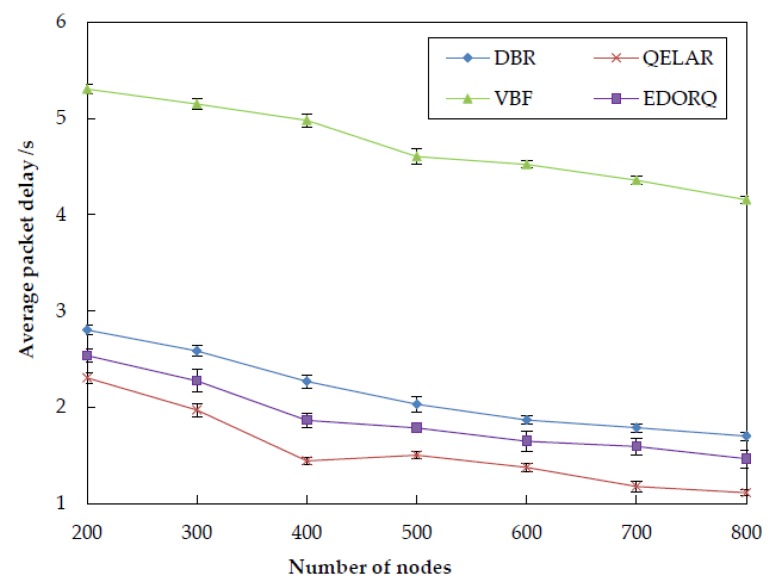
Average packet delay.

Figure 9 portrays the impact of node density to the average packet delay of the four different algorithms. We can see that the average packet delay of the four algorithms decreases with the increasing of the number of nodes, because the forwarding node can find more qualified nodes to relay packets in its neighborhoods. Another observation from Figure 9 is that the average packet delay of VBF is higher than other three algorithms. This can be explained by the fact that, in VBF, the packets are delivered only within the routing “pipe” formed from source to sink. However, the nodes located in the “pipe” may not be closer to the surface sink, resulting in higher average packet delay. The average packet delay of DBR is shorter than VBF, it is mainly because that DBR utilizes the multiple-sinks architecture while VBF has only one fixed sink on the water surface. The performance of our proposal in terms of average packet delay is better than DBR but a litter weaker than QELAR. Because the Q-leaning based candidate coordination method in EDORQ helps to find the global optimal next hop forwarder to reduce the packet delay, instead of a local optimal forwarder in DBR, it has a less average latency than DBR. Our method utilizes a holding time based on Q-value to coordinate the forwarding of candidate nodes, in which each packet needs wait for a moment before it be forwarded, thus causing a slightly higher average packet delay than QELAR.

**Figure 10:**
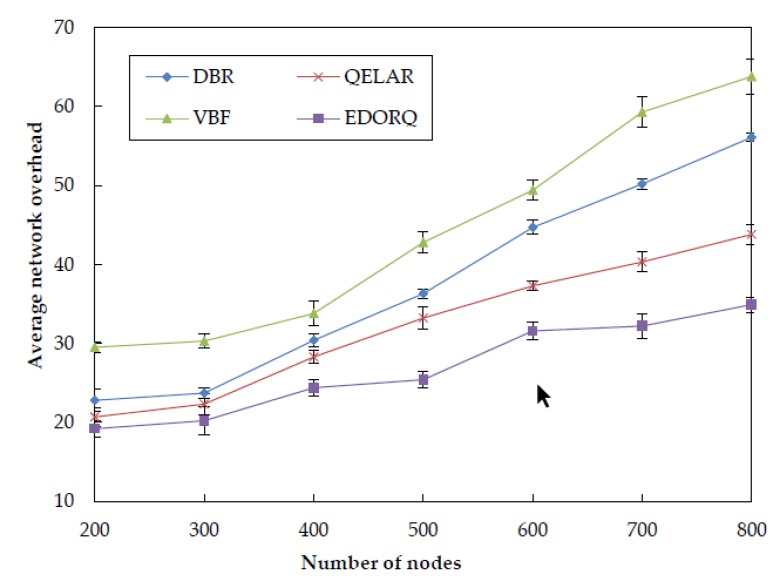
Average network overhead.

The results for the average network overhead of the four algorithms at different node densities are illustrated in Figure 10. We can observe that the average network overhead of DBR is smaller than VBF. The reason is that, in VBF, there are too many sensor nodes are contributed to the process of data forwarding without effective redundant packets suppression technique. Compared to VBF, the utilizing of depth threshold and timer in DBR greatly reduces the number of redundant copies in the network, resulting in the reduction of average overhead. QELAR causes many overhead in the initial stage of routing process when using the Q-learning technique. However, it can find a path of the length near the shortest one, therefore the overall overhead is smaller than DBR. Compared with QELAR, the proposed EDORQ algorithm applies the timer-based candidate set coordination mechanism based on Q-learning, which decreases the number of packet retransmissions. Meanwhile, it exchanges the related information of Q-value in only one hop neighbor without too many extra control packets, thus it suppresses redundant packet transmissions and avoids causing too much extra overhead. Therefore, the proposed scheme attains superior performance in average network overhead to other three algorithms.

#### 5.3.2. Impact of Sink Number

**Figure 11:**
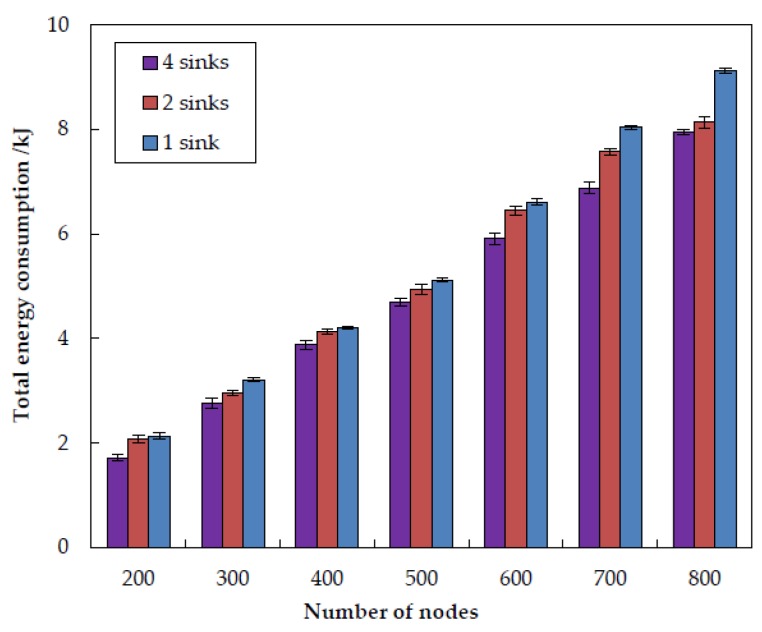
Impact of different number of sinks on the total energy consumption.

**Figure 12:**
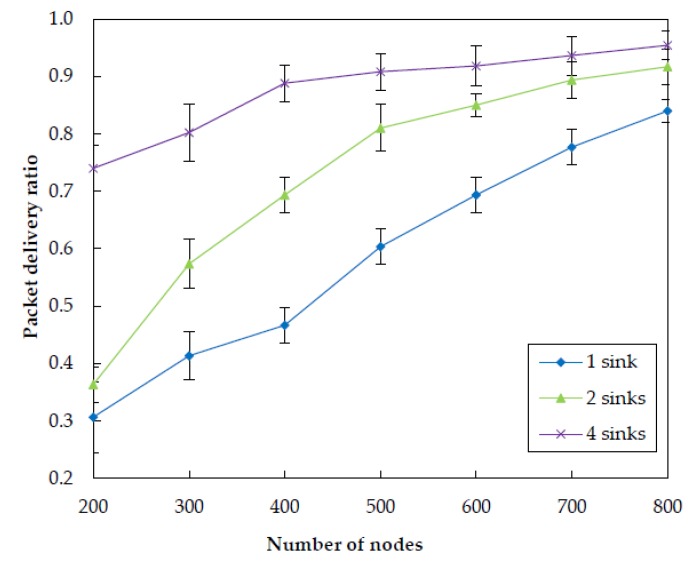
Impact of different number of sinks on the packet delivery ratio.

**Figure 13:**
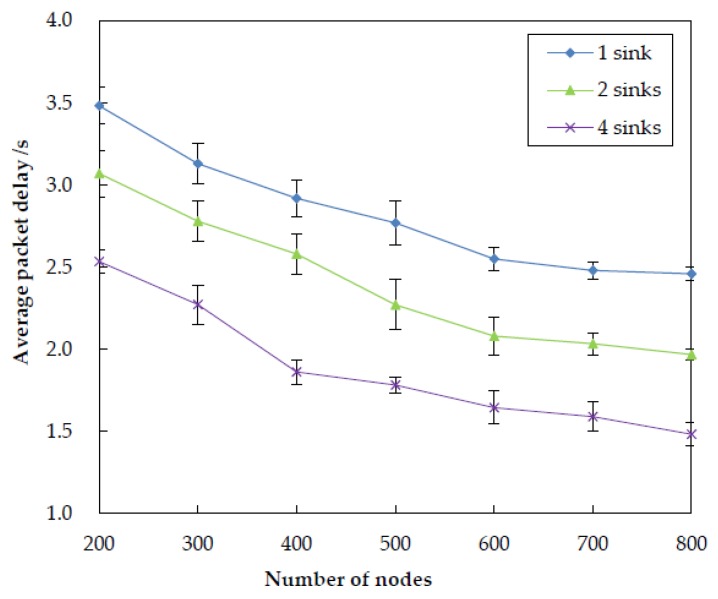
Impact of different number of sinks on the average packet delay.

**Figure 14:**
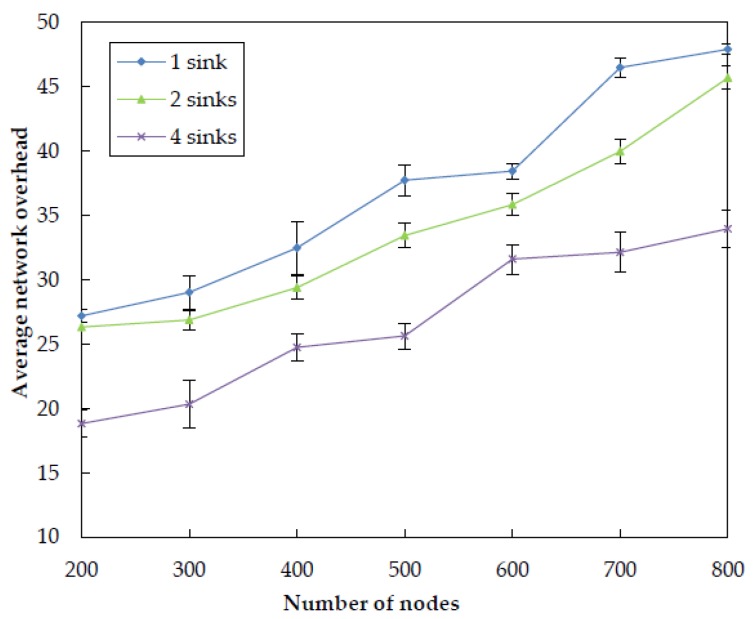
Impact of different number of sinks on the average network overhead.

In order to examine how number of sinks impacts the performance of EDORQ, we conduct extensive simulations at varied sink numbers of 1, 2 and 4 under the same operational condition as before. The simulation results for total energy consumption, packet delivery ratio, average packet delay and average network overhead are shown in Figure 11, Figure 12, Figure 13 and Figure 14, respectively. 

From the figures, we can observe that EDORQ has a lower total energy consumption, average packet delay and average network overhead while a higher packet delivery ratio with more the number of sinks. It is mainly because that, the fewer of the number of sinks, the greater the probability for a node close to the water surface to be a void node. Thus, with fewer sinks, the void recovery mode will be triggered more frequently to recover the data packet suffering from void nodes, which causes plenty of extra energy consumption, delay and network overhead. On the contrary, more sinks mean an increase of the chance for packets to be delivered to a sink along a shorter path with fewer nodes, which is beneficial to reduce the energy consumption, overhead and latency. What is more, the increasing of the number of sinks enhances the opportunity for each forwarding node to select more qualified neighbor nodes for packet transmission to any of the sinks, which improves the packet delivery ratio. 

#### 5.3.3. Impact of Node Mobility

**Figure 15:**
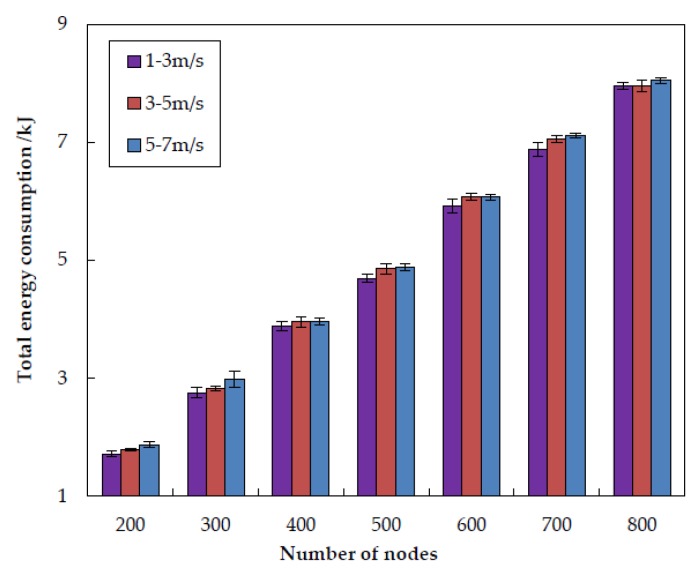
Impact of different node mobility on the total energy consumption.

**Figure 16:**
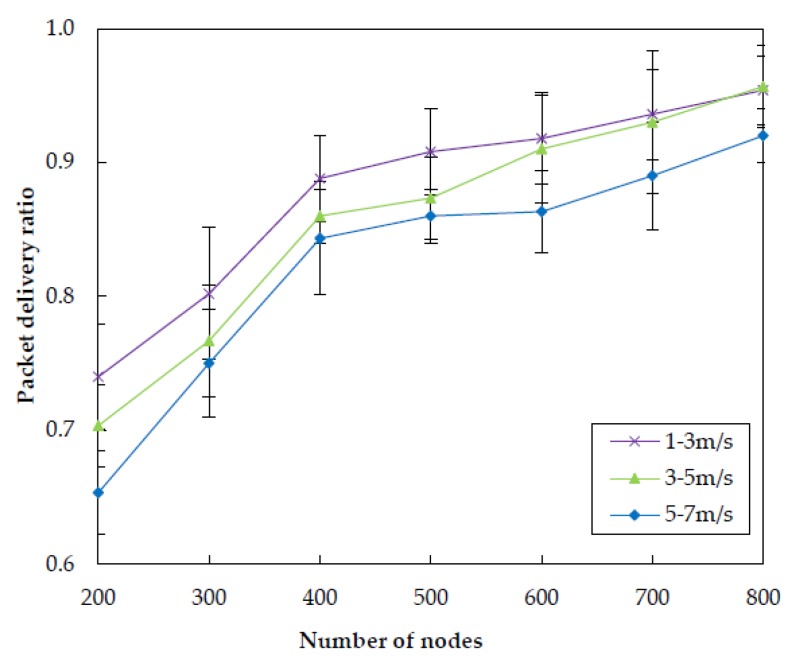
Impact of different node mobility on the packet delivery ratio.

**Figure 17:**
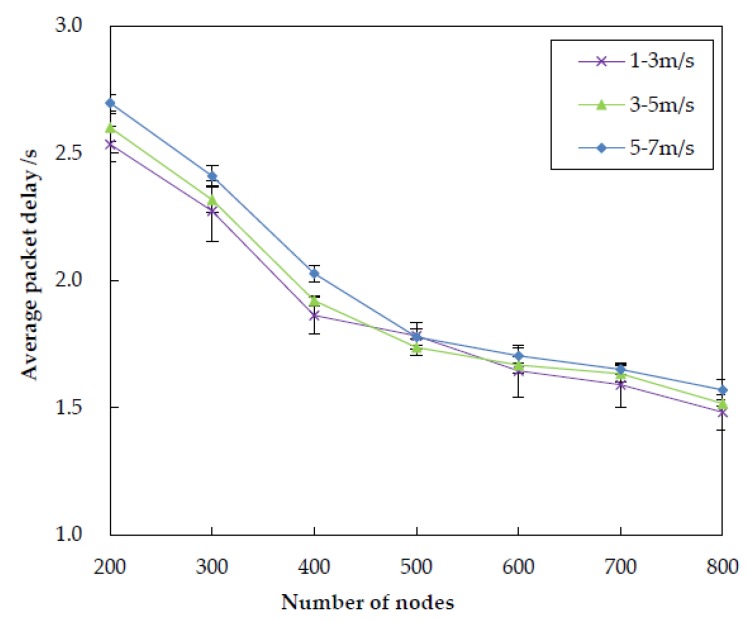
Impact of different node mobility on the average packet delay.

**Figure 18:**
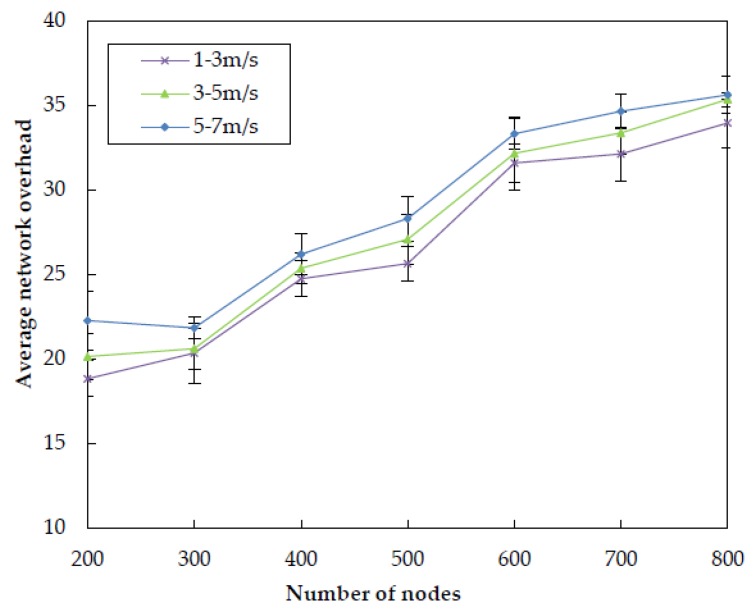
Impact of different node mobility on the average network overhead.

In this subsection, we investigate the impact of node mobility on the performance of EDORQ with different random speed intervals of [1, 3] m/s, [3, 5] m/s and [5, 7] m/s, respectively, and the other parameters are the same as Section 5.1. The first number in the bracket is the minimal speed and the second is the maximum speed. Each underwater node randomly selects a horizontal direction and moves with a random speed between the minimal speed and maximal speed. Figure 15, Figure 16, Figure 17 and Figure 18 show that how node mobility affects the network performance of EDORQ in terms of total energy consumption, packet delivery ratio, average packet delay and average network overhead, respectively. 

As can be observed from the figures, the total energy consumption, average packet delay and average network overhead will increase slightly while the packet delivery ratio will reduce slightly with the increasing of node speed. It shows that the movement of nodes within a certain speed range does not impact much on the performance of EDORQ algorithm. The reason is that, the OR features of our proposal can deal with the dynamic underwater topology to some extent by making the best of multiple potential forwarders. In addition, no exchange of topology or route information is involved among neighbor nodes in EDORQ. Furthermore, the Q-learning technique employed by EDORQ can learn from the network environment and make it have an adaptation to the dynamic underwater topology.

## 6. Conclusions

In this paper, we have proposed an energy-efficient depth-based opportunistic routing algorithm with Q-learning (EDORQ) for UWSNs to provide energy-saving and reliable data transmission, which improves the performance of network in terms of energy consumption, average network overhead and packet deliver ratio by combining the advantages of OR algorithm and Q-learning technique. It does not need the three-dimensional location information of sensors or other positioning method like ToA or AOA. In EDORQ, we design the direct reward function with a joint consideration of a void detection factor, residual energy and depth difference of sensor nodes to extend the Q-value, which can contribute to detecting and bypassing the void node in advance; meanwhile, minimizing the energy consumption. Furthermore, we design a void recovery mode in the candidate set selection phase to further recover the packet forwarding that is unfortunately trapped in the void nodes. What is more, we propose a novel holing time mechanism based on Q-value to further alleviate collisions and redundant forwarding. Simulation results show that, compared with the DBR [22], VBF [31] and QELAR [26] algorithms, our proposal significantly improves the performance in terms of energy efficiency, packet delivery ratio and average network overhead without sacrificing too much average packet delay. In the future, we will investigate the novel method to minimize the average packet delay of our proposed EDORQ to make it more flexible to many applications. Additionally, we intend to further extend the Q-value in our EDORQ for different research purposes by considering the influence of ocean currents, channel capacity and other performance indicators.

## Figures and Tables

**Table 1:** The main symbols used in the equations.

Name	Description
$\rho_{j}$	The void detection factor of node *n_j_*
*E_j_*	The energy-related factor of node *n_j_*
*D_ij_*	The depth-related factor between nodes *n_i_* and *n_j_*
$\delta_{ij}$	Binary-valued variable
α	Adjustment coefficients of $\rho_{j}$
β	Weight factor of rewards *E_j_* and *D_ij_*
$T_{j}$	The holding time of node *n_j_*

**Table 2:** Main Simulation Parameters.

Parameters Name	Value
Simulation scene range	500 m × 500 m × 500 m
*R*_max_	100 m
Send power	2 w
Receive power	0.1 w
Idle power	0.01 w
Discount factor γ	0.9
α	0.5
β	0.5
Simulation time for each run	800 s
